# The complete mitochondrial genome of the moss *Neckeropsis nitidula* (Mitt.) M. Fleisch.

**DOI:** 10.1080/23802359.2022.2055979

**Published:** 2022-04-03

**Authors:** Yeong-Deok Han, Young-Jun Yoon

**Affiliations:** Research Center for Endangered Species, National Institute of Ecology, Korea

**Keywords:** *Neckeropsis nitidula*, moss, mitochondrial genome

## Abstract

In this study, the complete mitochondrial genome of *Neckeropsis nitidula* (Mitt.) M. Fleisch. in the family Neckeraceae was determined. This mitogenome was 104,716 base pairs in length and contained 40 protein-coding genes, three ribosomal RNA genes, and 24 transfer RNA genes. The overall nucleotide composition was asymmetric (29.5% A, 29.3% T, 21.3% G, and 19.9% C), with a 41.2% G + C content. The gene arrangement was identical to that of most Hypnales genomes. A phylogenetic tree was constructed using the *N*. *nitidula* mitogenome obtained in this study and the complete mitochondrial genome sequences of 23 bryophytes and 3 Marchantiophyta that were publicly available in GenBank. Our phylogenetic analysis revealed that *N. nitidula* was clustered in a clade with other Hypnalean taxa.

The family Neckeraceae mostly consists of tropical and subtropical mosses. Sixteen genera are currently accepted worldwide (Crosby et al. 2000), of which five genera are found in South Korea (Park and Choi 2007). The genus *Neckeropsis* (Bryophyta, Neckeraceae) is represented in Korea by one taxon (Choe 1980). *Neckeropsis nitidula* [(Mitt.) Max Fleischer 1908] grows on tree trunks in lowland forests and on rocks, soil, and walls and is widely distributed in Korea, China, Japan, and Taiwan (Noguchi 1989). Morphologically, *N. nitidula* resembles *Homaliodendron microdendron*; however, it can be differentiated by its characteristic nonpinnately branched stems and the presence of double costae I n its leaves (Wu et al. 2011). In this study, the complete mitogenome of *N. nitidula* was determined using sequencing data, which may be helpful for future phylogenetic studies on diverse moss lineages.

A specimen of *N. nitidula* was collected from Gujwa-eup in Jeju-do (33° 29′ 2.01′' N 126° 48′ 0.12′' E) on 18 March 2021. The specimen was given voucher number YYJ 20210318-1 and stored in the Jeonbuk National University Herbarium(JNU) in Korea (Voucher Storage: Jeonbuk National University; Voucher number: YYJ 20210318-1; The person in charge of the collection: Y.-J. Yoon (email: liebejun@hanmail.net).

Genomic DNA was extracted from the fresh thallus material using DNeasy^®^ Plant Mini Kit (QIAGEN, Hilden, Germany), and then was prepared to genomic library using QIAseq FX Single Cell DNA library kit (QIAGEN). Genome sequencing was performed using HiSeq (paired-end mode, 150 bp) (Illumina, San Diego, CA, USA), and the raw sequencing reads (about 5 GB) were trimmed and quality checked using Trimmomatics v0.36 (Bolger et al. 2014) and FastQC v0.11.5 (Andrews 2010), respectively. The mitogenome sequence of *N. nitidula* was assembled using the NOVOplasty 2.4 and Geneious v8.1.9 software, with partial sequence of NADH dehydrogenase subunit 5 of *N. nitidula* as the seed sequence (Kearse et al. 2012; Dierckxsens et al. 2017). Genome annotation was conducted with reference to the mitogenomes of seven Hypnales species used in the phylogenetic tree using Geneious v8.1.9. The complete mitogenome sequence of *N*. *nitidula* was deposited in the National Center for Biotechnology Information GenBank database under the accession number MZ662079.

A total of 182,082 reads were used to assemble the mitogenome of *N*. *nitidula* with 251.8× mean coverage. The *N*. *nitidula* mitogenome had a length of 104,716 base pairs and consisted of 40 protein-coding genes (PCGs), three ribosomal RNA genes, and 24 transfer RNA genes (GenBank accession number MZ662079). The overall base composition was 29.5% A, 29.3% T, 19.9% C, and 21.3% G; specifically, the A + T content was 58.8% and the G + C content was 41.2%. All PCGs began with NTG start codons (ATG, CTG, and TTG) and ended with the typical termination codons (TAA, TAG, and TGA).

A concatenated data set used for the phylogenetic analysis was constructed with 33 PCG commonly included in the mitogenomes of 23 Bryophyta and 3 Marchantiophyta species registered in GenBank using Geneious v8.1.9. To determine the phylogenetic placement of *N*. *nitidula*, Bayesian phylogenetic tree was constructed with MrBayes 3.1.2 software based on GTR + I + G model by jModeltestv 2.1.10, and with 1000 bootstraps replicates. *Neckeropsis nitidula* was identified, albeit with no support, as a sister to *Anomodon rugelii* and *A. attenuatus*, and together with *Myuroclada*, *Hypnum*, *Climacium*, and *Myurella*, it is a part of the robust order Hypnales (Figure 1). The mitogenome of *N*. *nitidula* is the first of the family Neckeraceae in Korea to be assembled and may provide a reference for future phylogenetic studies of diverse moss lineages.

**Figure 1.**
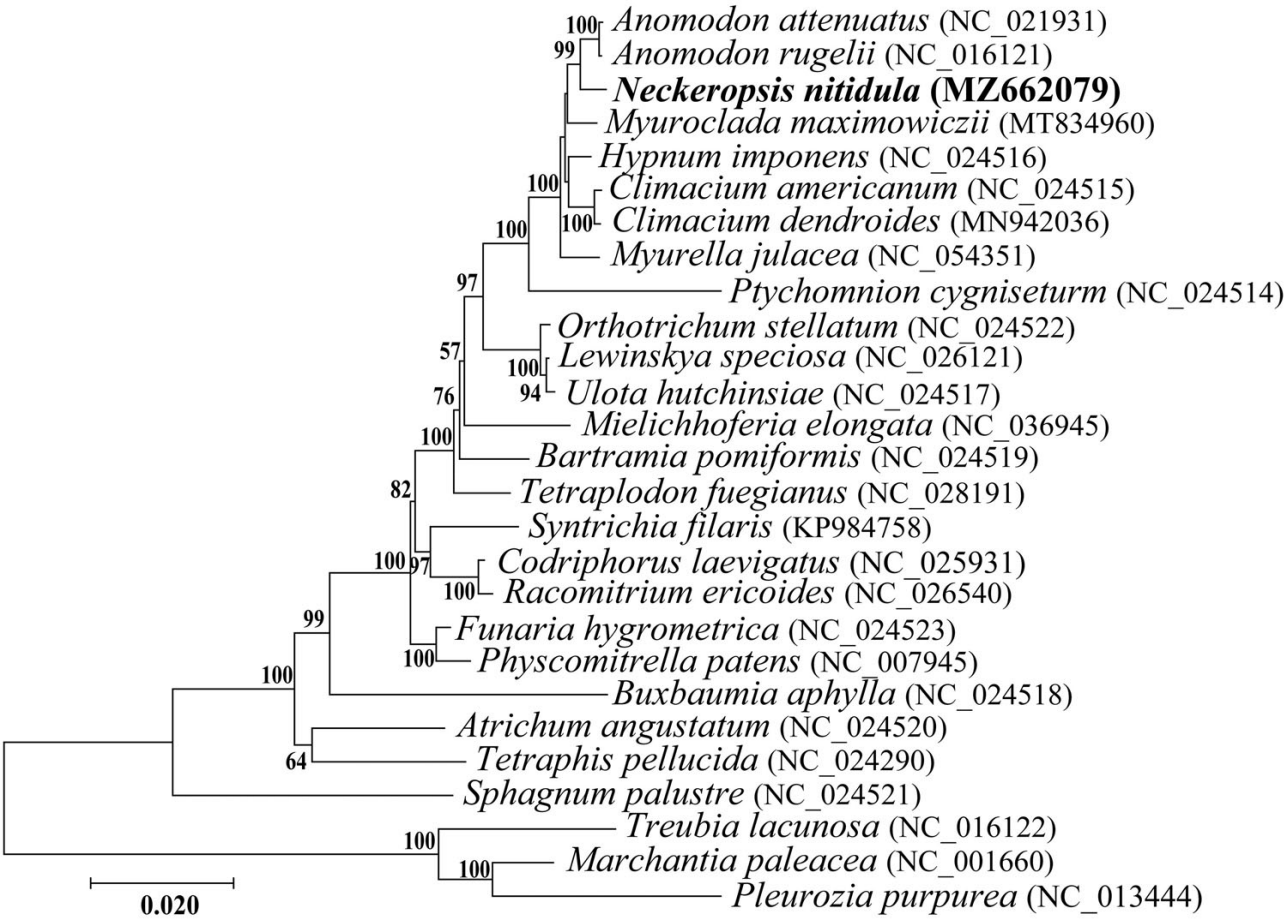
Phylogenetic relationship among *Neckeropsis nitidula* constructed by maximum-likelihood analysis based on 33 mitochondrial protein-coding genes common in all taxa. A bootstrap values above 50% are given at the nodes. Three Marchantiophyta species were selected as outgroup.

## Ethical approval

This study did not involve endangered or protected species, and the plant was collected under special permission from National Institute of Ecology. Field studies have been carried out in accordance with guidelines and comply with local legislation. One individual was collected from Gujwa-eup in Jeju-do (33° 29′ 08.64′' N 126° 48′ 30.37′' E), South Korea.

## Data Availability

The complete mitogenome sequence can be accessed via accession no. MZ662079 in GenBank of NCBI at https://www.ncbi.nlm.nih.gov. The associated BioProject, SRA, and Bio-Sample numbers are PRJNA749933, SAMN20426598, and SRR15264989, respectively.
